# Upregulation of programmed death-1 on T cells and programmed death ligand-1 on monocytes in septic shock patients

**DOI:** 10.1186/cc10059

**Published:** 2011-02-24

**Authors:** Yan Zhang, Jinbao Li, Jingsheng Lou, Ying Zhou, Lulong Bo, Jiali Zhu, Keming Zhu, Xiaojian Wan, Zailong Cai, Xiaoming Deng

**Affiliations:** 1 Clinical Research Center, Changhai Hospital, Second Military Medical University, 168 Changhai Road, Shanghai 200433, PR China; 2 Department of Anesthesiology, Changhai Hospital, Second Military Medical University, 168 Changhai Road, Shanghai 200433, PR China

## Abstract

**Introduction:**

Studies on the role of programmed death-1(PD-1) and its main ligand (PD-L1) during experimental models of sepsis have shown that the PD-1/PD-L1 pathway plays a pathologic role in altering microbial clearance, the innate inflammatory response and accelerated apoptosis in sepsis. However, the expression of PD-1 and PD-L1 and their role during the development of immune suppression in septic patients have not been elucidated. The present study was designed to determine whether the expression of PD-1 and PD-L1 is upregulated in septic shock patients and to explore the role of this pathway in sepsis-induced immunosuppression.

**Methods:**

Nineteen septic shock patients and 22 sex-matched and age-matched healthy controls were prospectively enrolled. Apoptosis in lymphocyte subpopulations and PD-1/PD-L1 expression on peripheral T cells, B cells and monocytes were measured using flow cytometry. Apoptosis of T cells induced by TNFα or T-cell receptor ligation *in vitro *and effects of anti-PD-L1 antibody administration were measured by flow cytometry. CD14^+ ^monocytes of septic shock patients were purified and incubated with either lipopolysaccharide, anti-PD-L1 antibody, isotype antibody, or a combination of lipopolysaccharide and anti-PD-L1 antibody or isotype antibody. Supernatants were harvested to examine production of cytokines by ELISA.

**Results:**

Compared with healthy controls, septic shock induced a marked increase in apoptosis as detected by the annexin-V binding and active caspase-3 on CD4^+ ^T cells, CD8^+ ^T cells and CD19^+ ^B cells. Expression of PD-1 on T cells and of PD-L1 on monocytes was dramatically upregulated in septic shock patients. PD-1/PD-L1 pathway blockade *in vitro *with anti-PD-L1 antibody decreased apoptosis of T cells induced by TNFα or T-cell receptor ligation. Meanwhile, this blockade potentiated the lipopolysaccharide-induced TNFα and IL-6 production and decreased IL-10 production by monocytes *in vitro*.

**Conclusions:**

The expression of PD-1 on T cells and PD-L1 on monocytes was upregulated in septic shock patients. The PD-1/PD-L1 pathway might play an essential role in sepsis-induced immunosuppression.

## Introduction

Sepsis, a systemic inflammatory response to infection, kills more than 210,000 people in the United States annually [1] and remains one of the most challenging clinical problems worldwide, constituting the leading cause of death in noncoronary intensive care units (ICUs) [2].

Sepsis initiates a complex immunologic response that varies over time with the concomitant occurrence of both proinflammatory and anti-inflammatory mechanisms alternatively predominating. After a short proinflammatory phase, septic patients enter a stage of protracted immunosuppression, which is an important underlying cause of mortality during the late stage of sepsis. This immunosuppression in sepsis is clinically manifest by cutaneous anergy, hypothermia, leucopenia, susceptibility to infection, and failure to clear infection [3-5].

Monocytes play an essential role in the innate immune defense against microbial infection. Septic immunoparalysis is first characterized by a monocytic deactivation of phagocytic function, proinflammatory cytokine release, and antigen-presenting capacity (probably due to a decreased expression of HLA-DR) [6,7]. Importantly, the persistence of immunoparalysis, is correlated with an increased risk of fatal outcomes [8]. On the other hand, accumulating evidence points to the pivotal role of increased immune effector cell apoptosis in sepsis-induced immunosuppression [9,10]. Uptake of apoptotic cells by macrophages and dendritic cells (DCs) stimulates immune tolerance by inducing the release of anti-inflammatory cytokines, including IL-10 and transforming growth factor beta, and suppressing the release of proinflammatory cytokines. Inhibition of lymphocyte apoptosis can improve survival in animal models of sepsis by using selective caspase inhibitors [11,12], by altering proapoptotic/antiapoptotic protein expression [13,14], and by treatment with survival-promoting cytokines such as IL-7 [15] and/or IL-15 [16].

Sepsis produces marked alterations in the expression of membrane-associated co-stimulatory/inhibitory molecules. Expression of these accessory molecules appears to contribute to the morbidity/mortality seen not only in acute models of lethal septic challenge but in patients with septic shock [17,18]. Programmed death-1 (PD-1) is a newly identified co-inhibitory receptor. PD-1 has two main ligands--PD-L1 (B7-H1) and PD-L2 (B7-DC) [19]. PD-1 and its ligands exert inhibitory effects in the setting of persistent antigenic stimulation by regulating the balance between T-cell activation, tolerance, and immunopathology. The PD-1/PD-L1 pathway has been shown to be a crucial modulator of host immune responses in regulation of autoimmunity, tumor immunity, transplantation immunity, allergy, immune privilege, and ischemia/reperfusion injury [20]. Recent findings suggest that the PD-1/PD-L1 pathway plays an important role in the interaction between host and pathogenic microbes that evolved to resist immune responses. Those pathogens include viruses [21], certain bacteria [22], fungi [23], and some worms [24].

Some work has been carried out on the role of the PD-1/PD-L1 pathway in a model of sepsis, which showed that the pathway played a pathologic role in altering microbial clearance, the innate inflammatory response, and accelerated apoptosis in sepsis [25,26]. Huang and colleagues showed that PD-1 deficiency protects mice from the lethality of sepsis by balancing efficient pathogen clearance and inflammatory cytokine production [25]. Brahmamdam and colleagues showed that the administration of anti-PD-1 antibody 24 hours after cecal ligation and puncture (CLP)-induced sepsis prevented sepsis-induced depletion of lymphocytes and DCs, increased the expression of Bcl-xL, inhibited apoptosis, and improved survival, indicating that PD-1 blockade is a potential promising therapeutic target for sepsis [26]. Our recent work showed that expression of PD-1 on T cells, B cells and monocytes, and expression of PD-L1 on B cells and monocytes, were upregulated in septic mice compared with sham-operated controls. PD-L1 blockade significantly improved survival of CLP mice by preventing sepsis-induced depletion of lymphocytes, increasing TNFα and IL-6 production, decreasing IL-10 production, and enhancing bacterial clearance [27].

Brahmamdam and colleagues also showed that there is an increase of PD-1 expression on CD4 and CD8 cells after CLP-induced sepsis [26]. Huang and colleagues demonstrated that PD-1 expression on circulating monocytes was higher in patients with septic shock than in healthy volunteers [25]. Only five patients were included in the study, however, and the change of PD-1 expression in patients with sepsis was not the main objective in their study. In other words, the expression of PD-1 and PD-L1 and their role have not been elucidated in patients with sepsis. In this article, we present a cohort study designed to determine the expression changes of PD-1 and PD-L1 in septic shock patients.

## Materials and methods

### Patients and controls

The present study was conducted with approval from the ethics board of the Second Military Medical University, China. Patients were included after written informed consent signed by them or their next of kin. Nineteen consecutive patients with septic shock were prospectively included according to the diagnostic criteria of the American College of Chest Physicians/Society of Critical Care Medicine [28]. Patients were admitted to the surgical ICU of the Changhai Hospital, Second Military Medical University (Shanghai, China). The onset of septic shock was defined by the beginning of vasopressive therapy. The exclusion criteria included a lack of informed consent, age under 18 years, pre-existing hematological or immunological disease, and the absence of circulating leukocytes.

Patients were treated according to the standardized recommendations of our ICU. Arterial blood samples were obtained from each patient on the day of inclusion. After blood sampling in the ICU, tubes were transported at 4°C to the clinical research center within 2 hours for the measurement of apoptosis and expression of PD-1 and PD-L1. Flow cytometry staining was first performed as described below. The remaining blood was then processed to isolate peripheral blood mononuclear cells (PBMCs) by Ficoll density gradient centrifugation (within 3 hours) and CD14^+ ^monocyte purification. To provide panels of control values for flow cytometry analysis, 22 sex-matched and age-matched healthy individuals (age 58.6 ± 4.3 years; 11 females, 11 males) with no known co-morbidities were also included.

### Apoptosis measurements by flow cytometry

One hundred microliters of whole blood were subjected to VersaLyse lysing solution (Beckman-Coulter, Hialeah, FL, USA) for 15 minutes at room temperature. After washing, cells were incubated with phycoerythrin-labeled antibodies directed against CD4, CD8 and CD19. Apoptosis induction in each specific lymphocyte subpopulation - CD4^+ ^T cells, CD8^+ ^T cells and CD19^+ ^B cells - was assessed using annexin-V binding and intracellular active caspase-3 measurements.

Regarding the annexin-V binding experiments, according to the manufacturer's protocol, lysed samples were incubated for 15 minutes with phycoerythrin-labeled annexin-V (Annexin-V-PE Apoptosis Detection Kit; BD Biosciences, San Jose, CA, USA) and measured on a flow cytometer within 30 minutes using CellQuest software version 3.2 (BD Biosciences, San Jose, CA, USA). Results are expressed as percentages of respective cell populations positive for annexin-V binding. A threshold for positivity was set up based on nonstained controls.

For active caspase-3 intracellular staining, following two washes, lymphocytes were fixed and permeabilized using Cytofix/Cytoperm reagent (BD Biosciences) and were incubated with phycoerythrin-labeled anti-active caspase-3 antibodies (BD Biosciences). Isotype control antibodies were used to determine nonspecific binding. After one further wash, cells were analyzed by flow cytometry. Results are expressed as percentages of respective cell populations positive for caspase-3.

### PD-1 and PD-L1 expression on peripheral T cells, B cells and monocytes

Blood samples were obtained from 19 septic patients and 22 healthy controls. After erythrocytes were lysed using fluorescence-activated cell sorting lysing solution (BD Bioscience), cells were stained with fluorochrome-conjugated anti-CD3, anti-CD19, anti-CD14, anti-PD-1 or anti-PD-L1 antibodies. Flow cytometric analysis (50,000 events/sample) was performed on a FACSCalibur Flow Cytometer (BD Biosciences) using CellQuest software version 3.2. T cells, B cells or monocytes were gated on CD4^+^/CD8^+ ^cells, CD19^+ ^cells or CD14^+ ^cells, respectively.

Antibodies were purchased from eBioscience (San Jose, CA, USA): CD4-FITC (Clone RPA-T4, catalog number 12-0049), CD8-APC (Clone RPA-T8, catalog number 17-0088), CD19-PE-Cy5 (Clone HIB19, catalog number 15-0199), CD14-FITC (Clone 61D3, catalog number 11-0149), PD-1-PE (Clone MIH4, catalog number 12-9969), and PD-L1-PE (Clone MIH1, catalog number 12-5983).

### Induction of T-cell apoptosis and PD-L1 blockade *in vitro*

PBMCs were separated from whole blood of septic patients using standard gradient centrifugation with Lymphocyte Separation Medium (PAA Laboratories GmbH, Pasching, Austria) and were cultured at 4 × 10^5 ^cells/well in plates. Apoptosis was analyzed by flow cytometry after addition of 10 ng/ml TNFα (Peprotech, Rocky Hill, NJ, USA) alone or with anti-PD-L1 antibody (10 μg/ml, Clone MIH1, catalog number 16-5983; eBioscience) or isotype (10 μg/ml) for 18 hours. Alternatively, PBMCs were treated with 10 μg/ml anti-CD3 and 5 μg/ml anti-CD28 (eBioscience) alone or with anti-PD-L1 antibody (10 μg/ml) or isotype (10 μg/ml) for 72 hours. Cells were double-stained with annexin V and propidium iodide, with gating on CD3-positive cells, and were analyzed by fluorescence-activated cell sorting. Apoptosis was calculated as the percentage of annexin V-positive/propidium iodide-negative cells after gating on CD3-positive cells.

### Purification of human CD14^+ ^monocytes, lipopolysaccharide stimulation and PD-L1 blockade *in vitro*

PBMCs were separated from whole blood of septic patients and CD14^+ ^monocytes were purified using immunomagnetic beads coated with anti-CD14 monoclonal antibody (Miltenyi Biotec. Bergisch Gladbach, Germany) as previously described by Saikh and colleagues [29]. Flow cytometry analysis of the purified population demonstrated that more than 95% was positive for CD14 expression. CD14^+ ^monocytes were incubated with either lipopolysaccharide (100 ng/ml), anti-PD-L1 antibody (10 μg/ml), isotype antibody (10 μg/ml), or a combination of lipopolysaccharide (100 ng/ml) and anti-PD-L1 antibody (10 μg/ml) or isotype antibody (10 μg/ml). Twenty-four hours later, supernatant was harvested to detect cytokine production such as TNFα, IL-6, and IL-10 by ELISA according to the manufacturer's instructions (R&D Systems, Minneapolis, MN, USA).

### Statistical analysis

Data are reported as the mean ± standard error of the mean. All statistical analyses were carried out with Prism 4.0 (GraphPad Software, La Jolla, CA, USA). Comparisons between healthy controls and septic shock patients were made using the nonparametric Mann-Whitney U test with *P *< 0.05 considered statistically significant.

## Results

### Characteristics of the septic patient cohort

Nineteen patients with septic shock (nine women and 10 men) and 22 healthy volunteers (sex-matched and age-matched) were enrolled in the current study. The demographic and clinical characteristics of the cohort are presented in Table 1. None of the septic shock patients were previously immunocompromised (HIV, cancer, immunosuppressive treatments). Patients did not receive drotrecogin alfa (activated) before or during their treatment. Six patients received adjunctive corticosteroid treatment (3 mg/kg hydrocortisone) before or at the time of sampling. Nine septic shock patients died during their ICU stay.

**Table 1:** Demographic and clinical data for septic shock patients

Parameter	Patients (*n *= 19)	Healthy controls (*n *= 22)
Age at admission (years)	58 ± 4	59 ± 4
Gender (male)	10	11
APACHE II score at inclusion	26 ± 3	NA
SAPS II score at inclusion	55 ± 3	NA
Mechanically ventilated at inclusion (*n*)	16	NA
Antibiotic treatment at inclusion (*n*)	16	NA
Adjunctive corticosteroid treatment^a ^(*n*)	6	NA
White blood cell count at inclusion (g/l)	15.6 ± 2.8	6.5 ± 1.7
Lymphocyte count at inclusion (g/l)	0.92 ± 0.21	1.6 ± 0.8
Mortality (*n*)	9	0

Data presented as *n *or the mean ± standard error. APACHE, Acute Physiology and Chronic Health Evaluation; NA, not applicable; SAPS, Simplified Acute Physiology Score. ^a^Hydrocortisone, before or at the time of sample acquisition.

Underlying diseases of the septic shock group were necrotizing fasciitis (*n *= 3), fecal peritonitis (*n *= 9), and pneumonia (*n *= 7). The total number of leukocytes was increased in septic patients compared with healthy volunteers. In contrast, the total lymphocyte cell count was significantly diminished in shock patients compared with normal values.

### Annexin-V binding and caspase-3 activation measured by flow cytometry

**Figure 1:**
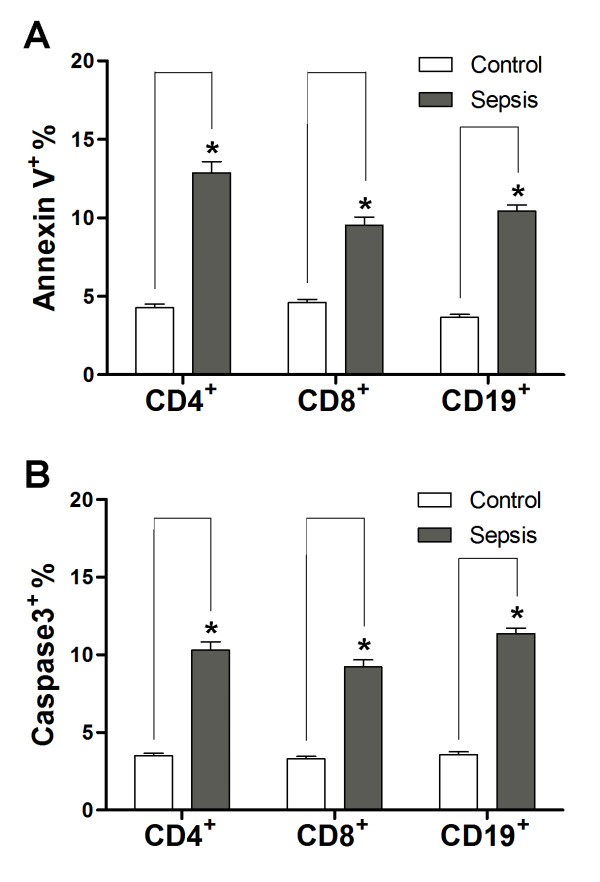
**Confirmation of accelerated apoptosis in septic shock patients**. **(a) **Annexin-V binding on CD4^+^, CD8^+ ^and CD19^+ ^lymphocytes of patients with septic shock. The population of annexin-V binding lymphocytes increased. *P < 0.01 compared with healthy controls. **(b) **Detection of active caspase-3 in lymphocyte populations. In patients with severe sepsis, the percentage of active caspase-3 positive lymphocytes (CD4^+ ^T cells, CD8^+ ^T cells, CD19^+ ^B cells) increased. *P < 0.01 compared with healthy controls.

We further assessed apoptosis (annexin-V binding and active caspase-3) by flow cytometry. We observed a significant increase of annexin-V binding on CD4^+ ^T cells, CD8^+ ^T cells and CD19^+ ^B cells in septic shock patients (Figure 1a).

Caspase-3 is the central executioner caspase. Activation of caspase-3 leads to degradation of multiple intracellular substrates and to the typical morphological features of classical apoptosis. In patients with septic shock, the subpopulation with active caspase-3 was elevated in CD4^+ ^T cells, CD8^+ ^T cells and CD19^+ ^B cells compared with healthy controls (Figure 1b).

### Expression of PD-1 and PD-L1 measured by flow cytometry

**Figure 2:**
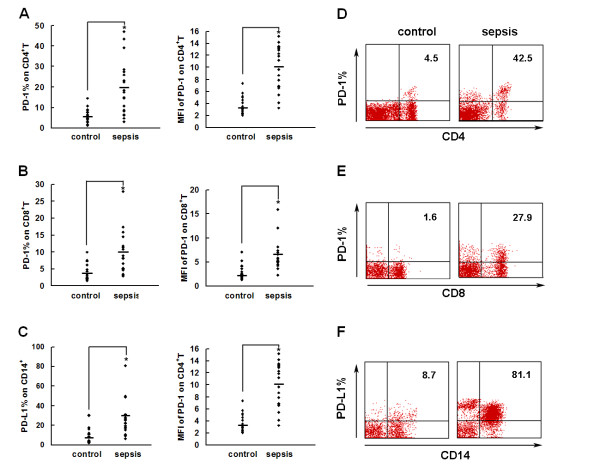
**PD-1 and PD-L1 were upregulated on T cells and monocytes in septic shock patients**. Blood samples were obtained from 19 septic shock patients and 22 healthy controls and were stained for programmed death-1 (PD-1) and programmed death ligand-1 (PD-L1) gated on CD4^+ ^T cells, CD8^+ ^T cells, and CD14^+ ^monocytes. **(a) to (c) **Percentage of PD-1 expression on (a) CD4^+ ^T cells and (b) CD8^+ ^T cells, and (c) percentage of PD-L1 expression on CD14^+ ^monocytes. Each dot represents one individual. Data are mean ± standard error of the mean (SEM) of three independent experiments. **P < 0.01 compared with healthy controls. **(d) to (f) **Mean fluorescence intensity (relative fluorescence units) of PD-1 expression on (d) CD4^+ ^T cells, (e) PD-1 expression on CD8^+ ^T cells, and (f) PD-L1 expression on CD14^+ ^monocytes Each dot represents one individual. Data are mean ± SEM of three independent experiments. *P < 0.05 compared with healthy controls. **(g)** Representative PD-1 expression levels on CD4^+ ^T cells and CD8^+ ^T cells, and PD-L1 expression on CD14^+ ^monocytes. Values in the upper-right quadrant indicate the percentage of cells that express PD-1 or PD-L1.

PD-1 and PD-L1 expression on T cells, B cells and monocytes in peripheral blood from septic patients was measured (Figure 2). The results showed that expression of PD-1 on both CD4^+ ^T cells and CD8^+ ^T cells in septic shock patients was much higher than that on healthy control cells (4.63-fold and 2.37-fold, *P *< 0.01) (Figure 2a, b). PD-1 expression was verified by the mean fluorescence intensity and showed similar results (*P *< 0.05) (Figure 2d, e). We also demonstrated that PD-L1 was dramatically upregulated on monocytes (3.58-fold, *P *< 0.01) compared with healthy subjects (Figure 2c, f) (*P *< 0.01). There was no change of PD-1 expression on either B cells (CD19^+^) or monocytes (CD14^+^). There was no change of PD-L1 expression on either T cells (CD4^+ ^and CD8^+^) or B cells (CD19^+^) (data not shown). Our data indicated that in addition to PD-1 upregulation on T cells, PD-L1 levels on monocytes also increased significantly.

### Effect of PD-L1 blockade on induced T-cell apoptosis *in vitro*

**Figure 3:**
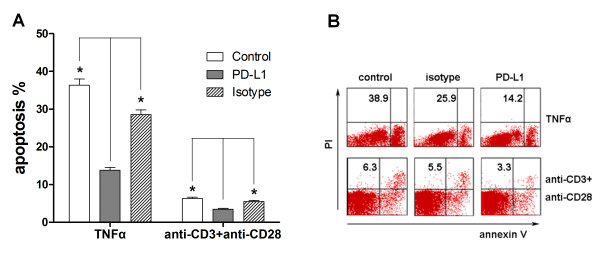
**Blockade of the PD-1/PD-L1 pathway by anti-PD-L1 antibody**. Blockade of the programmed death-1 (PD-1)/programmed death ligand-1 (PD-L1) pathway by anti-PD-L1 antibody decreased apoptosis of human peripheral T cells from septic patients induced by TNFα and by T-cell receptor ligation. **(a) **Peripheral blood mononuclear cells were obtained from septic shock patients and cultured at 4 × 10^5 ^per well in plates precoated with 10 ng/ml TNFα (Peprotech) alone or with anti-PD-L1 antibody(10 μg/ml) or isotype (10 μg/ml) for 18 hours to analyze apoptosis by flow cytometry. Alternatively, peripheral blood mononuclear cells were treated with 10 μg/ml anti-CD3 and 5 μg/ml anti-CD28 alone or with anti-PD-L1 antibody (10 μg/ml) or isotype (10 μg/ml) for 72 hours. Cells were doubled-stained with annexin V and propidium iodide (PI) with gating on CD3^+ ^cells and were analyzed by fluorescence-activated cell sorting. Apoptosis was calculated as the percentage of annexin V-positive/PI-negative cells after gating on CD3^+ ^cells. *P < 0.05 compared with control group. **(b) **Representative micrographs from six independent experiments. Representative data showing apoptosis of CD3^+ ^T cells.

To investigate the potential role of PD-1/PD-L1 on T-cell apoptosis in sepsis, we induced the apoptosis under stimulation with exogenous recombinant TNFα or anti-CD3 and anti-CD28 ligation. We found that PD-L1 blockade significantly induced a decrease of T-cell apoptosis. Cells treated with anti-PD-L1 antibody had an approximately 50% reduction in septic-shock-induced apoptosis in CD4 and CD8 T cells compared with the isotype control antibody-treated population in both TNFα-induced and T-cell receptor-induced apoptosis (Figure 3). Our results suggest that blockade of the PD-1/PD-L1 pathway could decrease human peripheral T-cell apoptosis.

### Effect of PD-L1 blockade on cytokine production of monocytes from septic shock patients *in vitro*

**Figure 4:**
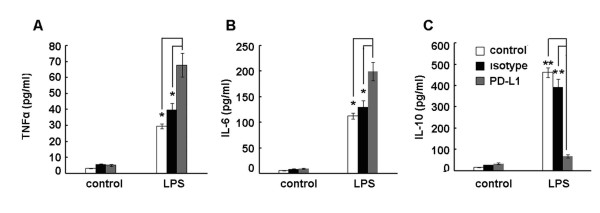
**Effect of anti-PD-L1 antibody treatment**. Anti-programmed death ligand-1 (anti-PD-L1) antibody treatment improved the ability of monocytes from septic shock patients to produce proinflammatory cytokines and decreased production of anti-inflammatory cytokines *in vitro*. **(a) to (c) **Peripheral blood mononuclear cells were separated from whole blood of septic patients and CD14^+ ^monocytes were purified using immunomagnetic beads coated with anti-CD14 monoclonal antibody. CD14^+ ^monocytes were incubated with either lipopolysaccharide (LPS) (100 ng/ml), anti-PD-L1 antibody (10 μg/ml), isotype antibody (10 μg/ml), or a combination of LPS (100 ng/ml) and anti-PD-L1 antibody (10 μg/ml) or isotype antibody (10 μg/ml) for 24 hours. The supernatants were collected for ELISAs of (a) TNFα, (b) IL-6 and (c) IL-10. Data are mean ± standard error of the mean of three independent experiments. *P < 0.05, **P < 0.01.

To assess the effect of the PD-1/PD-L1 pathway blockade on cytokine production of monocytes from septic shock patients *in vitro*, CD14^+ ^monocytes were isolated and purified from PBMCs and pretreated with anti-PD-L1 antibody or isotype control antibody before lipopolysaccharide stimulation. Supernatants were harvested after 24 hours to detect cytokine production by ELISA. We found that PD-L1 blockade enhanced the capacity of monocytes to produce proinflammatory cytokines, such as TNFα and IL-6 (Figure 4a, b), compared with isotype control antibody-treated cells. In contrast, IL-10 production was decreased significantly as compared with isotype control antibody-treated cells (Figure 4c). These results suggest that PD-1/PD-L1 pathway blockade by anti-PD-L1 antibody improved the function of monocytes isolated from septic patients.

## Discussion

The current study demonstrates that PD-1 on T cells and PD-L1 on monocytes are upregulated dramatically in a cohort of septic shock patients exhibiting accelerated lymphocyte apoptosis as compared with healthy controls. To our best knowledge, this is the largest number of patients specifically focused on to study PD-1 and PD-L1 expression in patients with sepsis. The patients with septic shock in the study exhibited accelerated apoptosis of all major lymphocyte subpopulations. The degree of apoptosis as indicated by annexin-V binding and caspase-3 activation was comparable with findings from previous reports [30-32]. The septic cohort of this study thus exhibited a degree of typical apoptosis of septic shock. PD-1 and its two known agonistic ligands, PD-L1 (B7-H1) and PD-L2 (B7-DC), are regulated by different mechanisms and are expressed in different cell types. Naïve T cells do not express PD-1, which is induced following engagement of the T-cell receptor. PD-1 remains expressed, however, on the surface of memory T cells. PD-L1 is present on multiple immune cells, including T cells and B cells, monocytes, DCs and macrophages, as well as on nonimmune cells, whereas PD-L2 expression is more restricted, present on activated DCs and macrophages [19,20]. Our study demonstrates that PD-1 on T cells and PD-L1 on monocytes are upregulated dramatically in septic shock patients. There was no change, however, of PD-1 expression on either B cells (CD19^+^) or monocytes (CD14^+^). There was no change of PD-L1 expression on either T cells (CD4^+ ^and CD8^+^) or B cells (CD19^+^).

Apoptosis of lymphocytes plays a pivotal role in immunosuppression. Multiple independent investigative groups have shown that the prevention of lymphocyte apoptosis improves survival in sepsis [11-16]. PD-1 and its ligand, PD-L1, deliver inhibitory signals that regulate the balance between T-cell activation, tolerance, and immunopathology. The PD-1/PD-L pathway has also been usurped by pathogens and tumors to attenuate antimicrobial or antitumor immunity, facilitating chronic infection and tumor survival. Recent work has been carried out on the role of the PD-1/PD-L1 pathway in a model of sepsis, which showed that PD-1/PD-L1 blockade is a potential promising therapeutic target for sepsis [25-27]. Blockade of PD-1 or PD-L1 results in enhanced T-cell responses, either through a direct pathway [33-35] or by abrogating the inhibitory function of regulatory T cells [36]. Herein, blockade of the PD-1/PD-L1 pathway decreased human peripheral T-cell apoptosis induced by TNFα and T-cell receptor ligation *in vitro*. Our data showed that PD-1 on T cells also appears to be an important mediator of T-lymphocyte apoptosis, which results in immunosuppression during sepsis.

Monocytes rapidly exhibit an impaired production of proinflammatory cytokines during sepsis although the underlying mechanism remains elusive [37]. In our study, we found dramatic upregulation of PD-L1 on monocytes from septic shock patients. *In vitro *PD-L1 blockade enhanced the capacity of monocytes from septic patients to produce proinflammatory cytokines, such as TNFα and IL-6, while decreasing the production of anti-inflammatory cytokines, such as IL-10. Our recent animal work showed that PD-L1 blockade increased TNFα and IL-6 production, and decreased IL-10 production in CLP mice. Taken together, we thought that the upregulation of PD-L1 on monocytes from septic shock patients might be associated with their functional decline, and thus may play an important role in immunosuppression [38]. These results also uncovered a role for PD-L1 that may be of great importance in the regulation of monocyte function seen during sepsis and may perhaps provide a novel mechanism underlying impaired monocyte function during sepsis.

Several limitations should be noted here. The overall sample size of the study is relatively small and we did not evaluate the changes of PD-1/PD-L1 expression over time after septic shock. This change in expression needs to be investigated in future studies. Another limitation is that the present study was not designed to predict the morbidity or mortality of septic shock, which are both also worth further investigation.

Taken together, our findings indicated that both PD-1 and PD-L1 were involved in sepsis-induced immunosuppression.

## Conclusions

Expression of the PD-1 on T cells and of PD-L1 on monocytes was upregulated in septic shock patients. Blocking the PD-1/PD-L1 pathway with anti-PD-L1 antibody resulted in decreased apoptosis of T cells and improved the ability of monocytes to produce proinflammatory cytokines *in vitro*. These novel findings suggest that the PD-1/PD-L1 pathway might be a useful target to treat sepsis-induced immunosuppression.

## Key messages

• Expression of PD-1 on T cells and of PD-L1 on monocytes was dramatically upregulated in septic shock patients.

• PD-1/PD-L1 pathway blockade *in vitro *with anti-PD-L1 antibody decreased apoptosis of T cells induced by TNFα or T-cell receptor ligation. Meanwhile, this blockade potentiated the lipopolysaccharide-induced TNFα and IL-6 production and decreased IL-10 production by monocytes *in vitro*.

• The PD-1/PD-L1 pathway might play an essential role in sepsis-induced immunosuppression.

## Abbreviations

CLP: cecal ligation and puncture; DC: dendritic cell; PD-1: programmed death-1; PD-L1: programmed death ligand-1; ELISA: enzyme-linked immunosorbent assay; HLA: human leukocyte antigen; ICU: intensive care unit; IL: interleukin; PBMC: peripheral blood mononuclear cell; TNF: tumor necrosis factor.

## Competing interests

The authors declare that they have no competing interests. The present work was partially supported by grant 30971510 from the National Natural Science Foundation of China.

## Authors' contributions

YZha, JBL and JSL contributed equally to the article; they all participated in the study design, collected blood samples, detected all of the samples by flow cytometry and ELISA kits, and also helped to analyze the data and draft the manuscript. YZho and JLZ helped to design the experiment, analyze the data, and draft the manuscript. LLB, XJW and KMZ helped to analyze the data. Both ZLC and XMD designed the experiment, supervised all of the experimental work and statistical analysis, and wrote the manuscript. All authors read and approved the final manuscript.
